# Identifying project topics and requirements in a citizen science project in rare diseases: a participative study

**DOI:** 10.1186/s13023-022-02514-3

**Published:** 2022-09-14

**Authors:** Michaela Neff, Holger Storf, Jessica Vasseur, Jörg Scheidt, Thomas Zerr, Andreas Khouri, Jannik Schaaf

**Affiliations:** 1 Institute of Medical Informatics, Goethe University Frankfurt, University Hospital, Frankfurt am Main, Germany; 2 Institute for Information Systems, University of Applied Sciences Hof, Hof, Germany

**Keywords:** Rare diseases, Citizen science, Patient science, Qualitative research

## Abstract

**Background:**

Due to their low prevalence (< 5 in 10,000), rare diseases are an important area of research, with the active participation of those affected being a key factor. In the Citizen Science project “SelEe” (Researching rare diseases in a citizen science approach), citizens collaborate with researchers using a digital application, developed as part of the project together with those affected, to answer research questions on rare diseases. The aim of this study was to define the rare diseases to be considered, the project topics and the initial requirements for the implementation in a digital application.

**Methods:**

To address our research questions, we took several steps to engage citizens, especially those affected by rare diseases. This approach included the following methods: pre- and post-survey (questionnaire), two workshops with focus group discussion and a requirements analysis workshop (with user stories).

**Results:**

In the pre-survey, citizens suggested 45 different rare diseases and many different disease groups to be considered in the project. Two main project topics (A) “Patient-guided documentation and data collection” (20 votes) and (B) “Exchange of experience and networking” (13 votes) were identified as priorities in the workshops and ranked in the post-survey. The requirements workshop resulted in ten user stories and six initial requirements to be implemented in the digital application.

**Conclusion:**

Qualitative, citizen science research can be used to collectively identify stakeholder needs, project topics and requirements for a digital application in specific areas, such as rare diseases.

**Supplementary Information:**

The online version contains supplementary material available at 10.1186/s13023-022-02514-3.

## Background

In the European Union, the prevalence of a rare diseases (RD) is defined as affecting no more than 5 in 10,000 persons. There are more than 6000 known different RDs, of which almost 72% caused by genetic abnormalities [1, 2]. Low prevalence, complex symptomatology, limited expertise, and lack of available health services require special efforts to obtain a specific and correct diagnosis and appropriate treatment [3–5].

As they face all these difficulties, patients with RDs and their relatives become experts on their disease. Therefore, even more than for common diseases, it is necessary to recognise them as informed and active project participants [6, 7]. People affected by a RD should therefore be directly involved in research projects, e.g. in Citizen Science (CS) projects, which engage people in the scientific process who do not work professionally in this field of research. Muki Haklay describes "participatory science", which includes CS, as the involvement of the population already in the formulation of the research question as well as in data collection [8].

The Project 'Seltene Erkrankungen bürgerwissenschafltich erforschen! (SelEe)' (engl. ‘Researching rare diseases in a citizen science approach’) is a joint CS project on RDs by the Institute for Information Systems at Hof University of Applied Sciences (iisys) and the Institute of Medical Informatics (IMI) at Goethe University Frankfurt. The project is funded by the Federal Ministry of Education and Research in Germany (BMBF) and supported by the Alliance of Chronic Diseases (ACHSE e.V.) [9, 10]. In SelEe, scientists and citizens aim to investigate RDs together by collecting data using a digital application. Citizens can contribute their knowledge and ideas directly to the project, formulate requirements, and improve collaboration between all stakeholders—starting with the initial phase of the project. During this phase, the challenges and problems in the daily lives of people affected by RDs—patients as well as their relatives (further referred to as ‘RD-affected persons’)—will be identified and addressed. In the context of the project, the term citizen also includes any interested non-scientists with no connection to RDs (further referred to as "interested persons"). The project will initially be carried out in Germany, with the possibility of a gradual international expansion.

To create a long-term benefit for all RD-affected persons, several steps were taken at the beginning of the project to identify topics that should be explored and implemented. The objectives of this study were to answer the following questions: (1) which RDs and groups of RDs should be considered, (2) which topics should be investigated for joint research on RDs using a digital application, and (3) which requirements for the digital application are considered most useful.

## Methods

**Fig. 1:**
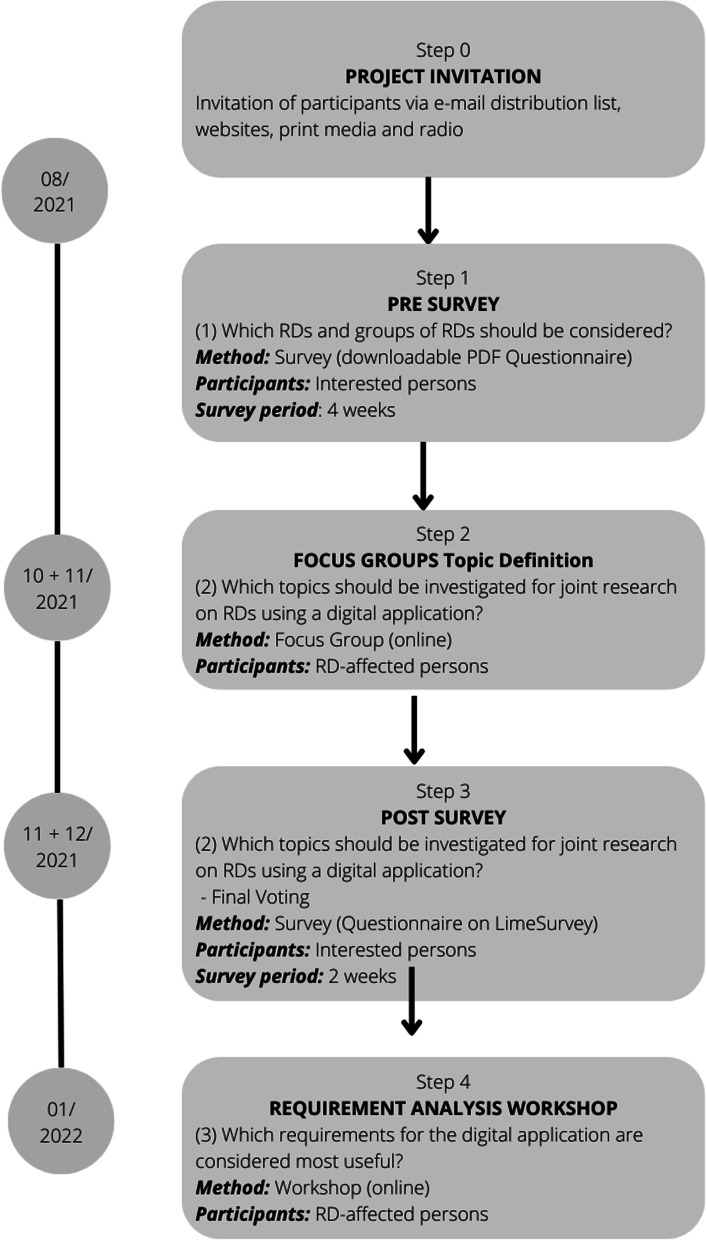
Steps of the study's multi-step approach

A multi-step approach was used to answer the research questions, including the following methods: questionnaires, focus groups and a requirements analysis workshop. Figure 1 illustrates the steps of this study, which will be described in more detail in the following sections. The Standards for Reporting Qualitative Research (SRQR) guideline was considered for reporting the focus groups [11]. A checklist is available in Additional file 1.

### Pre-survey

The invitation to the project was disseminated via various media by ACHSE e.V. (e-mail distribution list) and the science communication department of Hof University of Applied Sciences (websites, newspapers and radio in the local area). Those interested in joining the study were asked to participate in a pre-survey by completing a questionnaire in PDF format available on the project website (www.selee.de) and returning it by e-mail or letter.

The questionnaire contained six semi-open and open-ended questions in German language (Additional file 2), covering two categories of questions:

1. Questions 1–3 (Q1–3): Background of the interested person,
2. Questions 4–6 (Q4–6): Proposal of diseases and disease groups with optional justification and first topic suggestions regarding the SelEe project.

The survey was conducted over four weeks in August 2021. Data analysis of the survey was conducted using Microsoft Excel. To assign participants to expertise in specific RD groupings (RD, not a RD, unclear), the named disease in Q3 was checked using orphanet nomenclature [12].

### Focus groups topic definition

After the pre-survey, two focus groups were conducted. These moderated group discussions were used to engage citizens in the decision-making process and to collect and discuss different facets of challenges and topics of RD-affected persons [13–15].

#### Setting and sampling

The participants of the focus groups were selected from those who had completed the pre-survey, based on one of the following inclusion criteria: affected by an RD according to the EU-wide definition of RD, has an unclear diagnosis, or relative of an affected person. After pre-selection according to the inclusion criteria, participants were randomly selected and distributed to the two focus groups until a maximum number of participants of 12 persons per focus group was reached [13, 15]. Finally, the participants were invited by e-mail.

#### Data collection

Prior to conducting the focus groups, all participants received and signed a consent form and were provided with information about the study (including information about the researchers). The focus groups were performed online via a video-conference application in October and November 2021. Each focus group lasted approximately 120 min and was held in German language.

**Fig. 2:**
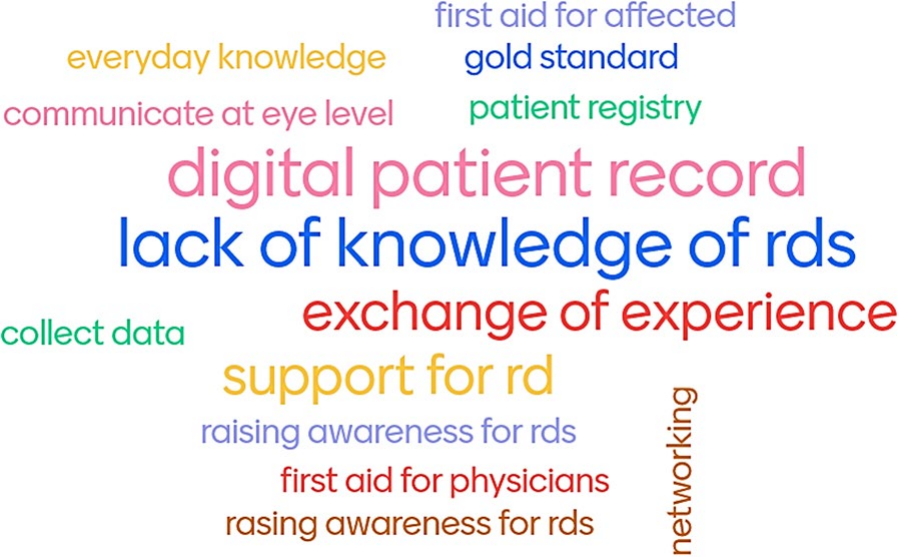
Interactive word cloud—which project topics would you like to propose?

A semi-structured interview guide (Additional file 3) was developed in preparation for the focus groups. In addition, an interactive word cloud online application [16] was used as a stimulus during the discussion (Fig. 2). First proposals for project topics were collected, initially showing suggested topics from Q6 of the pre-survey. The word cloud was then interactively updated and discussed by all participants.

Following a short round of introductions, the discussions during the focus groups were recorded via audio recording and moderated by two experienced female moderators from ACHSE e.V.. Two researchers from the SelEe project team created protocols of the project topic discussion to capture chat notes of the participants, visualize the topics in table form and prepare them for voting. Subsequently, all participants were asked to vote on the topics on a scale of 1 to 3 (1 = "most important", 2 = "very important", 3 = "important").

#### Data analysis and processing

The audio recordings were transcribed and reviewed independently by two researchers using the transcription system of Kuckartz et al. [17, 18]. The affiliation of the statements (participants/moderations team) were marked and the statements of the participants were anonymized. The transcripts were not distributed to the participants for correction or comments. However, participants received an anonymized summary of the results in German language. A translation of the quotations was made for the purpose of this publication.

Based on the transcript materials, the focus group protocols, and the results of the project topic ranking, central topics were identified. For this purpose, a content-structuring qualitative content analysis [19] was applied to combine the proposed topics from both focus groups and form categories to represent project topics. The main categories, including their sub-categories, which achieved the highest prioritization in the combination of both focus groups (taking into account the average of the voting of topics) were prepared for the post-survey.

### Post-survey

To identify a final project topic, a post-survey was conducted. In terms of CS, this survey was conducted as a follow-up questionnaire to the focus group with an expanded group of participants and was thus sent to all citizen (RD-affected persons and interested persons) in the project who had completed the pre-survey (Sect. 2.1), excluding those who had by then revoked their participation in the project.

The survey was conducted in anonymized form using the online tool LimeSurvey [20]. Repeated participations were ruled out using a dedicated feature of LimeSurvey. The survey was distributed via e-mail in November 2021 and was conducted over two weeks in November/December 2021. In the questionnaire, each participant had the opportunity to vote for exactly one project topic (Additional file 4). Data analysis for the survey was conducted using Microsoft Excel.

### Requirement analysis workshop

After establishing the project topic, a workshop was performed together with RD-affected persons to define specific requirements of the digital application for the implementation of the project topic. In this study, a requirement was defined as a software function that could be used by a user in a software system. The participants of both previous focus groups (Sect. 2.2) were invited as the designated primary user group of the digital application. The invitation was sent in January 2022 via e-mail. All participants again received and signed a consent form and further information before workshop participation.

The workshop lasted 120 min and started with a short presentation on the topic. Afterwards, user stories, visualised with story cards, were collected interactively and common requirements were discussed. A user story is an informal, general explanation of a software feature written from the end user's perspective [21]. Participants were advised to share their suggestions using the following user story template of Mike Cohn [22–24]: As <role> I want <goal, functionality> so that <some reason, benefit>.

The user stories were visualised and documented in Microsoft PowerPoint by two researchers and were visible to all participants. The common requirements were also noted visibly for the participants and documented in the researchers' notes. They were transferred from the researchers' notes into Microsoft Word.

## Results

### Pre-survey

The pre-survey conducted during participant recruitment was answered by 69 candidates, with affected persons and relatives making up the majority of participants. A breakdown of participants by group (Q1), previous experience (Q2), and knowledge of RDs through different backgrounds (Q3) is provided in Table 1.

**Table 1:** Experience and background of the participants (Q1- Q3)

Item	Frequency
*Q1: Group of participants (multiple answers possible)*	
Affected persons and relatives	68
Students	3
Medical professionals	3
Interested citizens	6
Others	6
No response	0
*Q2: Previous experience of participants (multiple answers possible)*	
Experience in the subject field of rare diseases	42
Scientific work	18
Statistics	10
Design and creation	5
Computer science	5
Citizen science	4
Others	16
No response	10
*Q3: Expertise in RD gained by (multiple answers possible)*	
Being affected by an RD	56
Being relative of a affected person	9
Studies	6
Profession	5
Others	3

The evaluation of the disease expertise given in the free text (Q3) in terms of classification as RD, no RD or unclear diagnosis is shown in Table 2.

**Table 2:** Evaluation of stated RD expertise (free text)

Evaluation of stated RD expertise	Frequency
Rare disease	49
Not a rare disease	16
Still unclear (unclear diagnostics)	4

In the optional question Q4, 20 combinations of disease groups were suggested for the project (Additional File 5). With the exception of the group 'Transplantation in Children', every disease group was mentioned at least once, with the following four groups accounting for almost half of the mentions:

- Immunodeficiency, autoinflammatory and autoimmune diseases
- Neurological diseases
- Neuromuscular diseases
- Rare multisystemic vascular diseases

In the optional question Q5, 45 different RDs were suggested as distinct diseases to be included in the project. The justifications (Q6) ranged from personal experiences to specific research gaps. A listing of the specific diseases (Q5) as well as the corresponding justifications for the suggestion (Q6) is not provided in this publication for reasons of personal reference (data privacy) e.g. for diseases with a very low prevalence.

### Focus groups topic definition

**Fig. 3:**
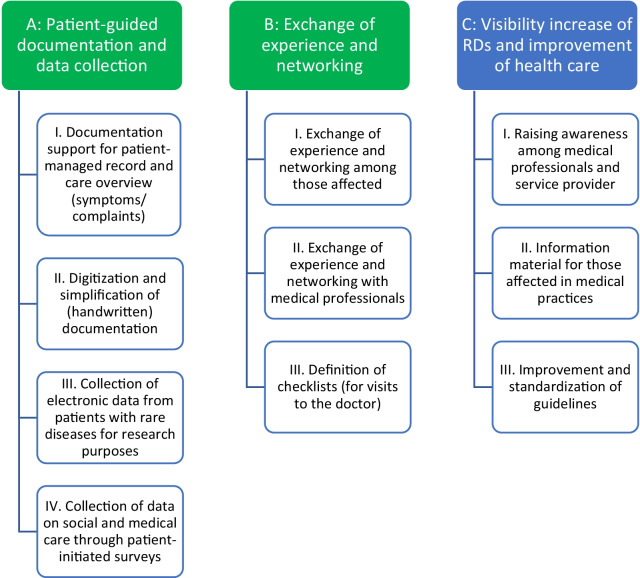
Results from category formation

The results of the focus groups (first focus group: 11 participants, second focus group: 9 participants) are presented below, organized by categories. The qualitative content analysis identified three main categories with three to four sub-categories (Fig. 3). References for selected quotations are given for each statement (Additional file 6). Exemplary quotations and field/focus group notes are also listed, abbreviated as "S" (statement/quotation) and "N" (note), and numbered in ascending order (e.g. S1, N1).

#### Main category A: patient-guided documentation and data collection

##### Documentation support for patient-managed record and care overview

The participants discussed that it would be helpful to provide a digital overview of the (social) care of RD patients in the digital application through the collection of receipts, doctor's letters and medical findings (S1, S2). Additionally, imaging results and the specific preparation of doctor's visits represent important aspects (S3). One participant described the preparation for a doctor's visit as follows:

Basically, every visit to the doctor is meticulously prepared so that we can bring the things exactly tailored to the request, […] and if I prepare everything well, then I have a good chance of getting my doctor’s prescription or my medical prescription. (S2)

Another participant stated that a translation function of the diagnostic findings would be useful when going abroad, especially a function that translates from German into English (S4). In addition, social aspects such as everyday life with severe RDs and paediatric patients as a subgroup in specific RDs were discussed (S5, S6).

Furthermore, electronic health records (EHRs) were declared inadequate in the discussion (S4, S7). Accessibility for people with disabilities, e.g. blindness, is often not considered in these applications (S2). Moreover, there were some statements by the participants on documentation support for symptom tracking. For RDs, there are often no adequate and customizable RD-specific applications to support digital documentation (S7, S9).

##### Digitization and simplification of (handwritten) documentation

As the digital applications currently available for documenting their information are inadequate (S2, S7, S9), patients use tools such as Microsoft Word or Excel to accurately record symptoms, diagnoses and medications with dates and times (S7, S8). One participant stated:

[…] So I sit down every three months and summarize that on an A4 sheet. There, again, I would like something, whether there are better options for recording. (S9)

##### Collection of electronic data from patients with rare diseases for research purposes

Patient registries have been discussed by the patients as tools to collect data on a specific RD for research purposes. Only a few registries are known to the participants and the question of including new diseases was raised (S10, S11). The collection of this data is an important factor for RD-affected persons and there is still a need:

[…] digital acquisition on a broad scale, which is also barrier-free for the visually impaired, for the blind, for the mobility-impaired or for the hearing-impaired, in whatever form. This will yield a much larger amount of data […] because the data are simply not available. If we patients can record this data, also in the respective quantities and with the respective accuracy, then there is a completely different foundation […] (S11)

##### Collection of data on social and medical care through patient-initiated surveys

One participant proposed a flexible survey instrument to cover medical and social aspects of patients with RDs:

[…] So that you have a tool to create quite flexible surveys. Maybe for patient organizations, so I would now like to invite everyone who has this syndrome or to investigate how they are doing, what support they need, how they organize their everyday life. […]. So, from an IT perspective, a flexible tool for surveys, and a way to reach people with rare diseases […]. (S5)

#### Main category B: exchange of experience and networking

##### Exchange of experience and networking among those affected

The communication among affected needs to extend beyond current disease-specific communities, e.g., through social media. Furthermore, participants discussed negative experiences with those communities (S8, S15, S16). One participant stated:

[…] I think the challenge will be to develop something that covers the non-specific in general. That's why I was thinking a bit about communities within this platform. Because that already works quite well on Facebook, Facebook groups, for specific diseases. But there, again, the general aspect is missing. (S8)

##### Exchange of experience and networking with medical professionals

Participants suggested better communication and more exchange with medical professionals e.g., through training initiated by patient organizations (S12).

##### Definition of checklists (for visits to the doctor)

Participants suggested checklists, as support for doctor visits. Similar approaches have been developed by RD patient organizations in the past. Moreover, some pharmaceutical companies also offer checklists, e.g., to rate specific symptoms (S3, S13, S14).

#### Main category C: visibility increase of RDs and improvement of health care

##### Raising awareness among medical professionals and service provider

For raising awareness of RDs, qualification of medical professionals in the field of RDs as well as improved financial possibilities, are desired by the participants (N1). Awareness of RDs should be strengthened, e.g., regarding the reimbursements of costs for treatment of RD patients (N2, N3, N4).

##### Information material for those affected in medical practices

The availability of information material for affected persons was addressed. One participant stated:

[…] It would be helpful, for example, if patient organization flyers could be displayed at the doctors’ offices […]. Where to find a patient organization? If the doctors were open to it […] I think you would also reach the people. (S15)

##### Improvement and standardization of guidelines

Furthermore, participants mentioned that clinical guidelines for RDs should be improved and standardized [N5].

### Prioritizing topics

In the first focus group 'Overview of the previous (social) care' from main category A achieved the highest prioritisation with an average of 1.0 (corresponds to “most important”). In the second focus group 'Exchange of experience and networking' from the main category B was prioritised with an average of 1.25. The complete table with all topics and results of the prioritisation during the focus groups, as well as the assignment to the categories, can be found in Additional file 7.

### Post-survey

The invitation was sent by e-mail to 63 prospective participants, of whom 33 of responded (response rate 52%). Due to the anonymity of the survey, no further information about the participants is available.

**Fig. 4:**
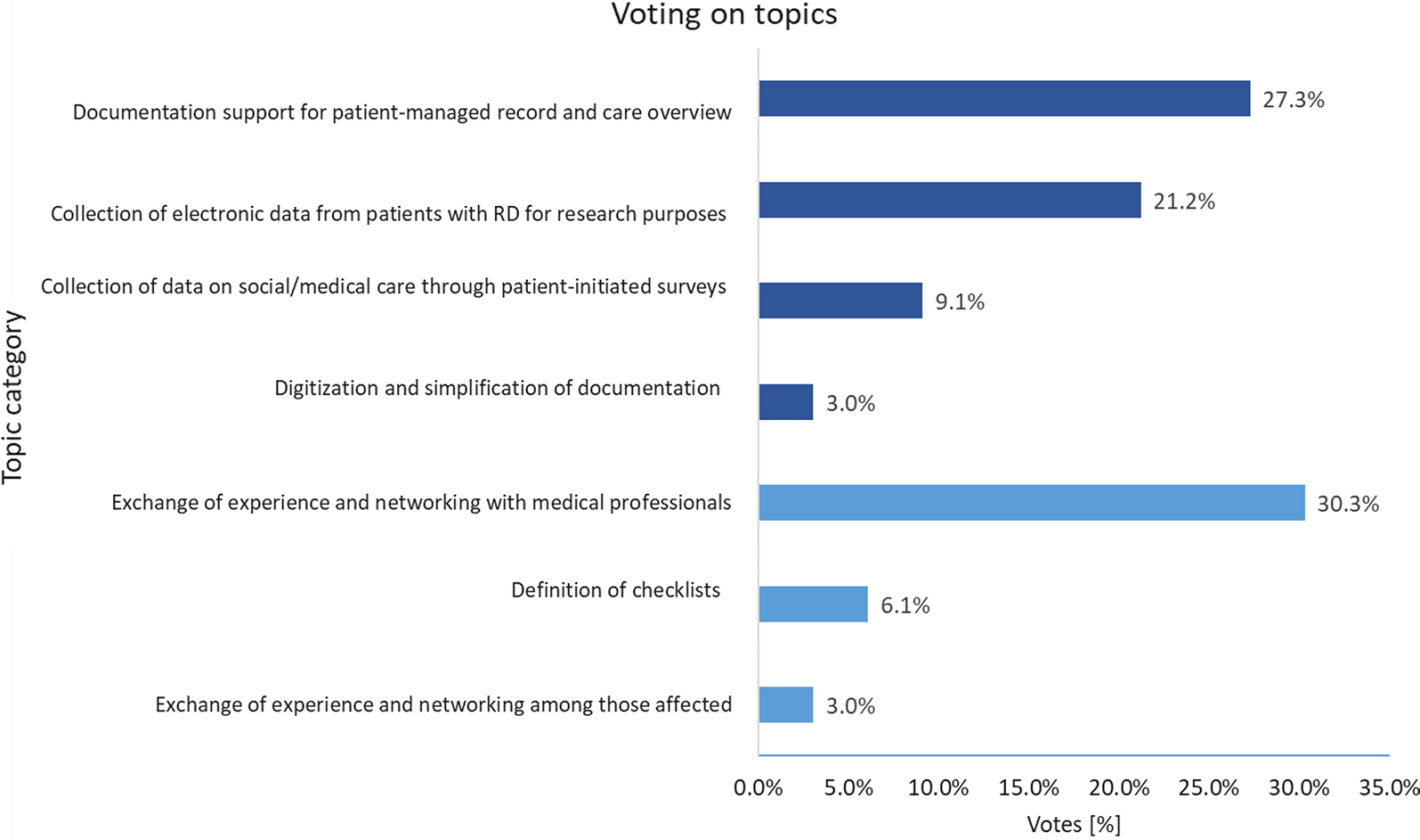
Voting on topics in post-survey

The results of the post-survey show that a total of 61% of the votes were received for main category A “Patient-guided documentation and data collection” and 39% of the votes for main category B “Exchange of experience and networking”. An overview of the voting is shown in Fig. 4.

### Requirement analysis workshop

**Fig. 5:**
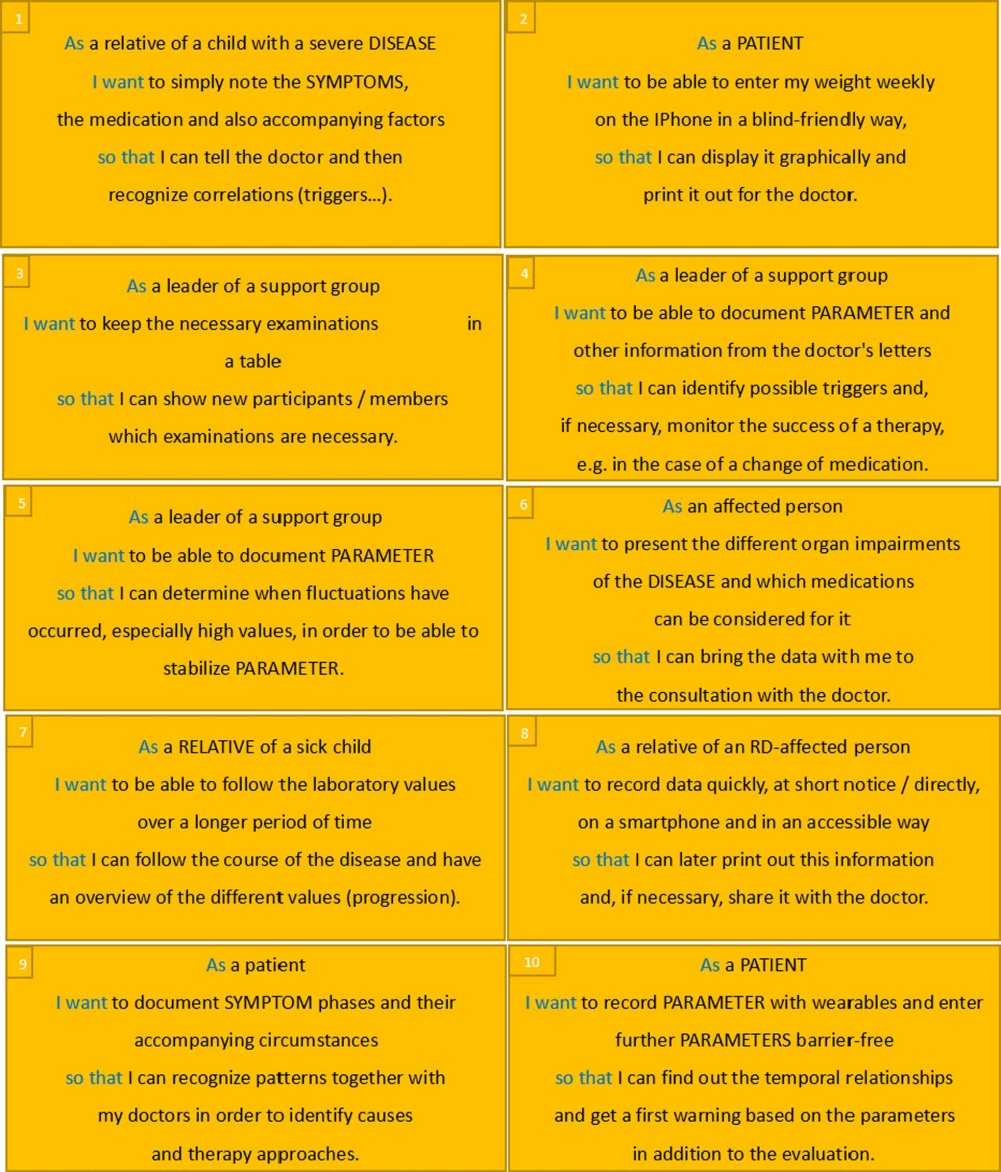
Story cards of RD-affected persons

Ten of the 19 invited participants attended in the workshop. They were able to define 10 user stories (visualised as story cards), which are shown in Fig. 5. If they referred to specific RDs, this information was anonymized for data protection reasons (anonymous terms are capitalised e.g. PATIENT).

Six common requirements for the digital application were defined, as shown in Table 3.

**Table 3:** Requirements for the SelEe digital application

No	Requirement	Description
1	Daily and retrospective data acquisition	Simple, accessible entry of daily updated data (e.g., health status, experiences, symptoms, medication) as well as retrospective data (e.g., laboratory results, doctor's letters)
2	Documentation of one or multiple symptom(s)	Possibility of documenting ≥ 1 symptom at regular intervals, using a configurable template that defines the parameter to be recorded
3	Visual representation of data	Graphical display of data (e.g., symptoms), e.g., as a representation of the long-term trend
4	Printout of documentation	Possibility to print the recorded documentation as a report
5	Export of data	Export of the collected data in various digital formats, preferably in a format that is easy to use for medical professionals
6	Support in recognizing correlations and patterns (together with medical professionals)	Possibility to recognize patterns and correlations in the symptoms, e.g., that one parameter always occurs at a certain time interval after another parameter. This should be enabled by a compact visual representation of the parameters e.g., by plotting parameters together over time. Interpretations should be possible by the RD-affected person together with medical professionals

## Discussion

### Overview

The motivation for this study was to define the project objectives and topics of the SelEe project, which should be implemented by using a digital application. This study offers insights into the challenges and needs related to RDs and provides ideas for a digital application that might offer direct added value to RD affected people.

### Discussion of methods

CS is often interpreted and implemented in different ways. There are a variety of approaches and no generally accepted definition [25, 26]. Particularly, there is still limited literature and best practices on the methodology of involving citizens in medical (informatics) projects [27, 28], especially in the context of RDs [29]. Heyen et al. published initial recommendations in a previous CS project in the field of RDs, which were taken into account in the study design [30]. In addition multi-step approaches for defining a digital application through user-centred design (UCD) have already been implemented in CS in other domains [31] and considered for this project.

The study design of SelEe is based on the mentioned considerations, as well as on specifically described criteria of the established methods of focus groups, qualitative content analysis and user stories [13, 17, 24]. However, focus groups and workshops conducted in a virtual format have shown benefits in terms of diversity of participants and reaching less healthy populations who are unable to travel [32, 33]. They therefore represent a promising option for this project.

In summary, the methodological approach of this study can be adopted by other researchers who want to develop digital applications in a specific area of healthcare and (medical) informatics by addressing the needs of stakeholders not previously considered.

### Discussion of results

The results of the pre-survey showed a wide range of suggested disease groups and distinct RDs, as well as the need for further research in the field of RDs. Despite the broad spectrum, participants described similar experiences in their justifications for the suggestion, which can already be found in the literature [34], e.g. in the EURORDIS list [7]. Based on these findings, SelEe will not focus on a specific RD or disease group but intends to address the common challenges mentioned by the study participants. The idea is to collaboratively develop and provide a digital application for data collection, using selected RDs as specific examples. As exemplary diseases, the project will focus on RDs from the TOP4 of the pre-survey and cooperate with corresponding patient organisations. In this case, the project is highly dependent on the participation of citizens in the further process. The adaptability of the digital application for additional RDs will ensure a view of all RDs and their commonalities.

With regard to the second research question, the following project topic was formulated as a result of the focus groups and confirmation through the post-survey: ‘Documentation support for a patient-managed record, including an overview of medical and social care and providing a basis for exchange and networking with medical professionals’. The proposed topic could address the problems already mentioned, such as the lack of information and scientific knowledge due to insufficient data and research [34] and facilitate data collection on many RD patients.

Following the topic definition, the requirements workshop allowed to consider the third research question. In addition to the individual user stories, overarching requirements were defined. The most important function of the digital application is the daily and retrospective recording of parameters (e.g. from a diagnostic report), which can be selected or additionally defined for the specific RD by those affected. According to our research and knowledge, we are not aware of any digital application that implements our project topic and offers the developed functionalities across several different RDs. Currently existing CS systems are placed in other fields of human medicine [10]. Regardless of the CS character, disease-specific apps can be found [35–41], apps and websites that refer people to health care providers [42, 43], apps for sharing experiences and networking [44, 45], a symptom checker [46], information and support apps for RD-affected persons [47, 48]. Some of these apps are available in English only.

In the next phase of SelEe, the gathered requirements will be further developed in close collaboration with citizens and will serve to answer research questions in the field of RDs, which will be defined collaboratively. Further studies are needed in the course of the project to investigate these questions, as well as the added value of the digital application for RD-affected persons. Following an initial data collection in the DACH region (Germany, Austria, Switzerland), the project idea is to be expanded to Europe and beyond, e.g. in cooperation with the European Citizen Science Association and EURORDIS. In this context, data protection aspects of the individual countries, further language options and consent must be taken into account.

### Limitations

This study followed a qualitative approach, which refers to a specific target group (patients and relatives), deals with a specific topic area of RDs and is currently limited to Germany.

The involvement of any citizens such as interested persons is still limited in the initial phase of the project. In the following phase of SelEe, citizens can get involved and participate in different ways, e.g. in analysing the collected data.

## Conclusion

This study suggests that there remains a need for research in the field of RDs, many open challenges and a need for the development of digital support applications for RDs, especially in the overall consideration of commonalities and in common solutions for the support of RDs. The multi-step approach allowed gathering project topics and requirements to a digital application which can be used by patients with RDs.

## Supplementary Information

**Additional file 1**: SRQR guideline.

**Additional file 2**: Pre survey questionnaire.

**Additional file 3**: Focus group interview guideline.

**Additional file 4**: Post survey questionnaire.

**Additional file 5**: Suggested disease groups.

**Additional file 6**: Transcript citations and translation.

**Additional file 7**: Topic prioritization.

## Data Availability

The ethics approval and consent for this study preclude the sharing of the raw data.

## Abbreviations

ACHSE: Alliance of Chronic Diseases
BMBF: Federal Ministry of Education and Research
CS: Citizen Science
EHR: Electronic Health Record
IISYS: Institute for Information Systems
IMI: Institute of Medical Informatics
RD: Rare Disease
SRQR: Standards for Reporting Qualitative Research
SelEe: Researching rare diseases in a citizen science approach
UCD: User-Centred Design
