# Supplementary material for: Identifying project topics and requirements in a citizen science project in rare diseases: a participative study
Source: Orphanet J Rare Dis. 2022 Sep 14;17:357. doi: 10.1186/s13023-022-02514-3 (PMC9476337; doi:10.1186/s13023-022-02514-3)
Supplement: Supplementary file 2 — Additional file 2: Pre survey questionnaire. [file 13023_2022_2514_MOESM2_ESM.pdf]

Additional file 2: Pre-Survey Questionnaire

Item no.	Questions	Answer options	Question type
Q1	I would place myself in the following group:	• Affected persons and relatives<br>• Students (school, (technical) college, university)<br>• Medical professionals<br>• Interested citizens<br>• Other	Multiple choice
Q2	I have experience, which I would like to contribute:	• Experience in the field of rare diseases<br>• Experience in citizen science<br>• Experience in scientific work<br>• Experience in statistics<br>• Experience in the field of design and creation<br>• Experience in computer science<br>• Other	Multiple choice
Q3	I have expertise on a specific rare disease/ on a group of diseases in the field of rare diseases:	• Yes: Open answer > 3a<br>• No	Single choice and open-ended question
Q3a	I have gained my expertise through:	• My job<br>• My studies<br>• I am affected by a rare disease<br>• I am a relative of a affected person	Multiple choice
Q4	I propose the following disease group for the project (optional):	• Endocrine diseases<br>• Hereditary metabolic disorders<br>• Childhood cancer<br>• Respiratory diseases<br>• Skin diseases<br>• Neuromuscular diseases<br>• Kidney diseases<br>• Neurological diseases<br>• Bone diseases<br>• Rare multisystemic vascular diseases<br>• Urogenital diseases<br>• Hepatological diseases	Multiple choice

		• Eye diseases<br>• Hematological diseases<br>• Congenital malformations and rare intellectual impairments<br>• Genetic tumor risk syndromes<br>• Transplantation in childhood<br>• Epilepsies<br>• Hereditary and congenital anomalies<br>• Craniofacial anomalies and ear, nose, throat disorders<br>• Heart diseases	
Q5	I suggest a certain disease for the project (optional):	Open answer	Open-ended question
Q6	Justification of the proposal (optional):	Open answer	Open-ended question
