# Supplementary material for: Identifying project topics and requirements in a citizen science project in rare diseases: a participative study
Source: Orphanet J Rare Dis. 2022 Sep 14;17:357. doi: 10.1186/s13023-022-02514-3 (PMC9476337; doi:10.1186/s13023-022-02514-3)
Supplement: Supplementary file 3 — Additional file 3: Focus group interview guideline. [file 13023_2022_2514_MOESM3_ESM.pdf]

Additional file 3: Focus group interview guideline

Guiding question, Story stimulus	Memo column (categories)	Questions to keep the conversation flowing
**What project topics would you like to propose?**	General issues, topics and challenges of rare diseases	• What challenges do you face?<br>• Where are there still open questions regarding rare diseases?<br>• Can you explain this with a concrete situation/example?<br>• What else would you add (word cloud shows first project suggestions of the participants)
**What are the most important issues for you?**	Prioritization	• What do these issues include? Can you explain this further?
**Do you have any experience with apps or websites and software, in general, to support you with your health care needs?**	Use, application possibilities and added value of digital application	• What would you recommend to your other patients?<br>• Do you keep a symptom diary or what do you do to prepare for a visit to the doctor or what and how do you regularly record data?<br>• I would still be interested to know what your experiences are here or where you would like to see more applications.
**What health data do you still document and how (on paper, with an app...)?**	Use of software tools for data collection	• What tools do you already know for documenting health data?<br>• Can you give some examples from everyday life?
**Do you have any ideas on how the challenges and problems mentioned could be addressed with the help of digital applications?**<br>**Do you have an idea what would support, but is not yet available in this way?**	How can research and digital applications support it?	
**Where do you still see open questions about your disease for research?**	Research ideas of the workshop participants	• Can you describe that in more detail?<br>• What do you mean by that exactly?
**We would now like to prioritize the mentioned research topics: from 1 to 3 (1="most important", 2="very important" and 3=" important")**	Topic prioritization	
**We have now reached the end of the workshop. Is there anything else we haven't talked about yet that is relevant to the topic?**	Thanking the participants and outlook for the	

	further procedure and timeline in the project	
