# Supplementary material for: Identifying project topics and requirements in a citizen science project in rare diseases: a participative study
Source: Orphanet J Rare Dis. 2022 Sep 14;17:357. doi: 10.1186/s13023-022-02514-3 (PMC9476337; doi:10.1186/s13023-022-02514-3)
Supplement: Supplementary file 4 — Additional file 4: Post survey questionnaire. [file 13023_2022_2514_MOESM4_ESM.pdf]

Additional file 4: Post survey questionnaire

Item no.	Questions	Answer options	Question type
Q1	Which of these topics is most important to you personally?	• Documentation support for patient-managed record and care overview (symptoms/complaints)<br>• Digitization of handwritten documentation (medical reports and own paper documentation<br>• Collection of electronic data from patients with rare diseases for research purposes<br>• Collect data on social and medical care through patient-initiated surveys (own survey tool)<br>• Exchange of experience and networking among those affected<br>• Exchange of experience and networking with medical practitioners<br>• Possibility to define checklists (for visits to the doctor)	Single choice
Q2	Explanation, questions and comments:	Open answer	Open-ended question
