# Supplementary material for: Identifying project topics and requirements in a citizen science project in rare diseases: a participative study
Source: Orphanet J Rare Dis. 2022 Sep 14;17:357. doi: 10.1186/s13023-022-02514-3 (PMC9476337; doi:10.1186/s13023-022-02514-3)
Supplement: Supplementary file 5 — Additional file 5: Suggested disease groups. [file 13023_2022_2514_MOESM5_ESM.pdf]

Additional file 5: Suggested disease groups

The following table shows the suggested disease groups for the SelEe project based on the pre-survey (question Q4, suggested group with the frequency of their mention)

Disease Group	Frequency
Immunodeficiency, autoinflammatory and autoimmune diseases	28
Neurological diseases	18
Neuromuscular diseases	15
Rare Multisystemic Vascular Diseases	11
Respiratory diseases	9
Haematological diseases	8
Endocrine conditions	8
Eye diseases	7
Diseases of the heart	6
Hereditary metabolic disorders	6
Skin disorders	6
Bone disorders	4
Hepatological diseases	4
Epilepsies	3
Adult cancers	2
Paediatric cancer (haemato-oncology)	2
Kidney diseases	2
Genetic tumour risk syndromes	2
Craniofacial anomalies and ENT disorders	2
Urogenital diseases and conditions	1
Inherited and congenital anomalies	1
Congenital malformations and rare intellectual disability	1
No Suggestion	9
