# Supplementary material for: Identifying project topics and requirements in a citizen science project in rare diseases: a participative study
Source: Orphanet J Rare Dis. 2022 Sep 14;17:357. doi: 10.1186/s13023-022-02514-3 (PMC9476337; doi:10.1186/s13023-022-02514-3)
Supplement: Supplementary file 6 — Additional file 6: Transcript citations and translation. [file 13023_2022_2514_MOESM6_ESM.pdf]

Additional file 6: Transcript citations and translation

Statement/ Citation	Original	Translation
S1	Seitdem ich meine Krankheit habe, habe ich alle Belege, ärztliche Befunde gesammelt. Vor allem wenn man zu einem neuen Arzt geht, dass man einfach für sich selber die Nachweise hat.	Since I have my disease, I have collected all the receipts, and medical reports. Especially when you go to a new doctor, you just have the supporting documents for yourself.
S2	Ja, Apps scheitern in der Regel an der Barrierefreiheit für MENSCHEN MIT BEHINDERUNG. Und ähnlich ist das auch mit der Software. Da ich noch nicht blindentechnisch rehabilitiert bin, und ich SPEZIELLE SOFTWARE noch nicht benutzen kann, wir versuchen es aber trotzdem. Also wir schreiben alles was ich trinke, was ich esse, die Medikamente, die ich nehme, die Symptome, die ich habe, kontinuierlich auf, mit Datum und Uhrzeit. Allerdings derzeit auf Papier. Und wir machen auch solche Sachen wie Messungen von Parametern wie z.B. Gewicht, Blutdruck und was man alles so hat und das diktiert mir meine ANGEHÖRIGE dann regelmäßig in Excel-Tabellen und fragt mich dann wo ich bin, und dann erstellen wir daraus auch Kurven für die Ärzte, damit diese auch den Verlauf beurteilen können. Jeder Arztbesuch wird minuziös vorbereitet im Grunde, damit man eben genau zugeschnitten auf das Anliegen die Sachen bringen kann, denn wir merken, dass die meisten Ärzte mit einer komplexen	Yes, apps usually fail because of accessibility for PEOPLE WITH DISABILITIES. And it is similar for software. Since I am not yet blind rehabilitated, and I cannot use SPECIFIC SOFTWARE yet, but we try anyway. So we write down everything I drink, what I eat, the medications I take, the symptoms I have, continuously, with dates and times. On paper, though, at the moment. And we also do things like measuring parameters such as weight, blood pressure and so on, and my RELATIVE regularly dictates this to me in Excel spreadsheets and asks me where I am, and then we use this to create curves for the doctors so that they can assess the progress. Basically, every visit to the doctor is meticulously prepared so that we can bring the things exactly tailored to the request, because we notice that most doctors are psychologically overwhelmed with a complex disease insofar that they first go on the defensive, and if I prepare everything well, then I have a good chance of getting my doctor’s prescription or my medical prescription.

	Erkrankung, psychologisch insofern überfordert sind, das sie erstmal in die Defensive gehen und wenn ich aber alles gut vorbereite dann habe ich genug Chancen, das ich dann auch meine Verordnung oder mein Rezept bekomme. Die Arztbriefe usw. werden natürlich alle aufgehoben und mit dem digitalisieren ist mit der fehlenden Barrierefreiheit für Blinde eben nicht weit her.	Of course, the doctor's letters, etc. are all kept and digitizing them is not very accessible for the blind.
S3	Ich fungiere ja auch als Ansprechpartnerin für die ERKRANKUNG, vorwiegend im REGION und habe mir hierfür auch eigens erstellte Checklisten, also ein Word-Dokument angelegt, was ich dann auch nochmal aktualisiere, wenn jetzt in der Wissenschaft sich etwas Neues ergibt. Und so kann ich meine Leute, die dann zum ersten Mal in ein ERKRANKUNG-Zentrum reisen und gut vorbereiten welche Befunde sie in der handhaben sollen und dann wäre es vielleicht auch gut, wenn die Patienten so ein Tool hätten, das sie ihre Unterlagen in ein Verzeichnis hochladen kann was man dann auch individuell den Fachärzten freischalten kann, damit man nicht immer die ganzen Unterlagen selber sich besorgen muss, einscannen muss und dann wiederum den Arzt vorab übersenden soll. Bei uns ist es eben durch die ERKRANKUNG wichtig, dass man eben die Bildgebung hat.	I also act as a contact person for the DISEASE, mainly in the REGION, and for this purpose I have also created checklists, i.e. a Word document, which I update again when something new emerges in science. So I can prepare my staff who are travelling to a DISEASE centre for the first time and prepare them well for which findings they should handle, and then it might also be good if the patients had such a tool that allows them to upload their documents to a directory which can then also be individually accessed by the specialists, so that they do not always have to get all the documents themselves, scan them and then send them to the doctor in advance. In our case, it is important to have the imaging data because of the DISEASE. For the doctors, this is the decisive aspect it almost always fails due to the amount of data. So there it would be great to have a tool that would benefit both the patients and the specialists.

	Die ist für die Ärzte eben alles entscheidend, es scheitert eigentlich auch immer an den Datenmengen. Also da wäre es super, wenn man ein Tool hätte, das sowohl für die Patienten als auch für die Fachärzte dienlich wäre	
S4	In der elektronischen Patientenakte ist das ja schon ein bisschen angedacht, was auch vorher angesprochen wurde, dass man Befunde hochladen kann, die man dann jeweils den Ärzten zur Verfügung stellen kann. Was ich da wahnsinnig praktisch fände, wäre, wenn es eine Übersetzungsfunktion gäbe, damit man auch im Ausland auf diese Akten zugreifen kann. Oder das zumindest die Befunde auch auf Englisch übersetzt werden, was dann doch die meisten Leute können. Wir haben das Problem immer im Urlaub, wenn da dann irgendwas ist, ein Notfall. Wie erklärt man den Leuten dann was für eine Erkrankung vorliegt. Und Befunde hat man dann auf Deutsch dabei und damit kann Niemand etwas anfangen.	In the electronic patient file, this is already somewhat planned, which was also mentioned earlier, that findings can be uploaded, which can then be made available to the doctors. What I would consider incredibly practical would be if there were a translation function so that these files could also be accessed abroad. Or that at least the findings are also translated into English, which most people are able to understand. We always have this problem on vacation when there is something, an emergency. How do you explain to people what kind of disease you have? And then you have the reports in German and nobody can do anything with them.
S5	Genau das medizinische. Das war jetzt die medizinische Seite. Finde ich auch sehr interessant. Und mich interessiert auch noch so die soziale Seite. Also dass man ein Tool hat mit dem man ganz flexible Umfragen erstellen könnte. Vielleicht so an Selbsthilfegruppen, so ich möchte jetzt mal an alle die dieses Syndrom	Exactly the medical aspect. That was the medical side of it. I also find that very interesting. But I am also interested in the social side. So that you have a tool to create quite flexible surveys. Maybe for patient organizations, so I would now like to invite everyone who has this syndrome or to investigate how they are doing, what support they need, how they

	habe oder dazu aufrufen zu untersuchen wie es ihnen geht, welche Unterstützung sie benötigen, wie sie ihren Alltag gestalten. So etwas würde mich interessieren. Also so aus IT-Sicht ein flexibles Tool für Umfragen, und eine Vorgehensweise wie erreiche in denn die Leute mit den Seltenen Erkrankungen, möglichst natürlich mit meiner eigenen Seltenen Erkrankung. Um so auch Erfahrungsaustausch herbeizuführen.	organize their everyday life. I would be interested in something like that. So, from an IT perspective, a flexible tool for surveys, and a way to reach people with rare diseases, if possible, of course, with my rare disease. In order to also initiate an exchange of experience.
S6	Danke, ich wollte damit eigentlich auch schon den Übergang zu den digitalen Möglichkeiten machen. Weil die peadrischen Patienten bei ERKRANKUNG irgendwie noch so eine Untergruppe sind. Weil normalerweise eben die älteren Patienten eher ORGANprobleme bekommen, aber diese ORGANerkrankungen im Kindesalter schon sehr spezifisch sind. Und da sind aber nur sehr, sehr wenige Kinder betroffen. Und das auch so persönliche Gruppentreffen wahrscheinlich Reisen über ganz Deutschland erfordern würden. Und wir haben jetzt schon eine Telegram-Gruppe, aber das ist dann wiederrum auch wieder (...) Ich bin da die Moderatorin. Wenn da dann irgendwas nicht über mich läuft, dann ist es wieder alles gleich kaputt sozusagen. Da wäre einfach so eine Lösung schön und eventuell sogar mit der Möglichkeit irgendwie Daten zu	Thank you, I actually wanted to make the crossover to the digital possibilities with this as well. Because the pediatric patients with DISEASE are somehow still a subgroup. Because usually, older patients tend to have ORGAN problems, but these ORGAN disorders are already very specific in childhood. And only very, very few children are affected. Moreover, even such personal group meetings would probably require travel all over Germany. We already have a Telegram group, but that, again, is (...) I am the moderator there. If something does not go through me, then everything is broken again, so to speak. It would be nice to have a solution like that and maybe even with the possibility to collect data somehow to actually just learn something about the DISEASE.

	erfassen, um einfach was darüber zu lernen tatsächlich über ERKRANKUNG.	
S7	Ich habe den Versuch gestartet die EPA App zu nutzen, aber das ist ja noch ein reines Theater. Ansonsten führe ich doch klassisch eine Word-List und Excel Tabelle. Medikamente, Diagnosen, Symptome, Kopfschmerztagebuch. Relativ klassisch, weil die Apps, wie gerade schon gesagt wurde zum Teil krankheitsspezifisch genug sind aber auch nicht allgemein, um sie anpassen zu können.	I have started the attempt to use the EPA app, but that is still a pure struggle. Otherwise, I maintain a classic Word list and Excel spreadsheet. Medications, diagnoses, symptoms, headache diary. Relatively classic, because the apps, as was just said, are partly disease-specific enough but not general to be customizable.
S8	Mir ist gerade so durch den Kopf gegangen. Hier sind ja ganz viele Menschen mit unterschiedlichen Erkrankungen und dementsprechend unterschiedlichen Symptomen. Und ich glaube so eine App diegrundsätzlich Datensätze sammeln kann ist schon sinnvoll. Aber mir kam, ob es nicht Sinn machen würde, wie so einzelne Communities zu integrieren. Weil wenn ich jetzt die App allgemein für meine Symptome nutzen würde, aber trotzdem den Kontakt nur zu der Gruppe Menschen suchen möchte, die auch die Gleiche Erkrankung hat. Finde ich, denke ich (…). Ich glaube es wird eine Herausforderung eine App zu entwickeln zum Beispiel, oder eine Plattform, wie auch immer. Die das Allgemeine, genauso wie das spezifischere abdeckt. Weil wenn ich	I just thought about it. There are a lot of people here with different diseases and thus different symptoms. And I think an app that can basically collect data sets makes sense. But I wondered whether it would not make sense to integrate individual communities in this way. Because if I would now use the app in general for my symptoms but still want to seek contact only with the group of people who also have the same disease. I find that I think (...). I think it will be a challenge to develop an app for example, or a platform, whatever. That covers the general, as well as the more specific. Because when I think about what a collection of apps there already is, for example, for people with diabetes, they exist and each has its advantages and disadvantages, and I think the challenge will be to develop something that covers the non-specific in general. That's why I

	daran denke, was es schon für ein Sammelsurium an Apps, z. B. für Menschen mit Diabetes gibt, die gibt es und jede hat seine Vor- und seine Nachteile, und ich denke die Herausforderung wird etwas zu entwickeln, das Allgemein unspezifisch abdeckt. Deshalb habe ich so ein bisschen an Communities innerhalb dieser Plattform gedacht. Weil das funktioniert jetzt auf Facebook, Facebook-Gruppen krankheitspezifisch auch schon ganz gut. Aber da fehlt auch wieder der Allgemeine Aspekt.	was thinking a bit about communities within this platform. Because that already works quite well on Facebook, Facebook groups, for specific diseases. But there, again, the general aspect is missing.
S9	Also in der ERKRANKUNG natürlich der SYMPTOM Kalender, ich habe mal versucht eine App zu benutzen aber es ist immer ein Problem, wenn es so eine seltene ERKRANKUNG Form ist. Deshalb ganz schnöde handschriftlich, die ich dann irgendwie für den Arzt grafisch verdichte. Ich setze mich also alle drei Monate hin und verdichte das auf einem A4 Blatt. Auch da würde ich etwas wünschen, ob es da bessere Aufzeichnungsmöglichkeiten gibt.	So in the DISEASE, of course, the SYMPTOM calendar, I tried using an app once but it is always a problem when dealing with such a rare DISEASE form. Therefore, quite plain handwritten, which I then somehow graphically summarize for the doctor.<br>So I sit down every three months and summarize that on an A4 sheet. There, again, I would like something, whether there are better options for recording.
S10	Also bei ERKANKUNG gibt es formal sogar zwei universitäre Register. Eines für Erwachsene, eines für Kinder. Ich kann das nicht sagen, wie es bei den Erwachsenen läuft, aber bei den Kindern das ist relativ unbefriedigend gewesen. In der Vergangenheit war das ich in der	So in the case of DISEASE, there are actually formally two university-based registries. One for adults, one for children. I cannot say how it is with the adults, but with the children, it has been relatively unsatisfactory. In the past, this was based at the UNIVERSITY and is now supposed to be transferred to

	UNIVERSITÄT angesiedelt und soll jetzt nach ORT übergehen, aber ich habe einmal dort Daten hingeschickt und eigentlich sollte es jährliche follow-ups geben, das hat es aber nie gegeben. Aber so vom Verein selbst gibt es kein Register. Aber es gab immer mal Versuche Daten zu sammeln, wo es wohl relativ gute Response dazu gab.	PLACE, but I sent data there once and there was actually supposed to be annual follow-ups, but there never were. But from the association itself there is no register. But there were always attempts to collect data with a relatively good response.
S11	Ja hört man mich? (…) gut. Ich denke nach wie vor, das es sehr sinnvoll wäre gezielt Erhebungen zwischen den Patienten zu machen. Und was meine Problematik mit der BEHINDERUNG angeht. Das betrifft ja nicht nur meine Erkrankung, sondern sicher auch mehr. Und vom Vorgehen her betrifft die ganze Sache sowieso auch andere Erkrankungen. Also das Symptome bei Patienten gezielt selbst erfasst werden können, auch über längere Zeiträume und im Zusammenhang mit zum Beispiel was man gegessen hat und ob man sich angestrengt hat, was sonst gewesen ist, welche Medikamente man genommen hat. Und die digitale Erfassung im breiten Maße, die auch Barrierefrei ist für sehbehinderte, für Blinde, für mobilitätseingeschränkte oder für Hörgeschädigte, in welcher Form auch immer. Die wird eine viel größere Datenmenge bringen, die im Rückschluss zeigt, dass Erscheinungen, die eben ich habe,	Yes, can you hear me? (...) good. I still think it would be very useful to do specific surveys among the patients. And as far as my problem with DISABILITY is concerned. That does not only concern my disease, but certainly others. And in terms of procedure, the whole thing also concerns other diseases anyway. In other words, symptoms can be recorded specifically by patients themselves, even over longer periods of time and in connection with, for example, what they have eaten and whether they have exerted themselves, what else has happened, what medication they have taken. And digital acquisition on a broad scale, which is also barrier-free for the visually impaired, for the blind, for the mobility-impaired or for the hearing-impaired, in whatever form. This will yield a much larger amount of data that will show in conclusion that phenomena that I have, for example, something like blindness, in a neurodegenerative disease, but also in many other diseases, similar things can then be determined,

	z.B. sowas wie eben eine Erblindung, bei einer neurodegenerativen Erkrankung, aber auch bei vielen anderen Erkrankungen kann man ähnliche Sachen dann feststellen, dass diese Dinge auftreten, wann sie auftreten, in welchem Zusammenhang sie auftreten und was hilft. Wenn es überhaupt etwas ist, was helfen kann. Und das dadurch dann, dass diese Daten erfasst werden, die Anerkennung kein Problem mehr sein sollte. Denn im Moment wird vieles ja geleugnet, weil die Daten einfach nicht da sind. Wenn wir Patienten das erfassen können, auch in den entsprechenden Mengen und in der entsprechenden Genauigkeit, dann ist da ja eine ganz andere Grundlage, dass man sagen kann „oh ja“, diese Symptome treten auf bei dieser Krankheit.	that these things occur, when they occur, in what context they occur and what helps. If there is something that can help at all. And that by collecting this data, recognition should no longer be a problem. Because at the moment many things are denied because the data are simply not available. If we patients can record this data, also in the respective quantities and with the respective accuracy, then there is a completely different foundation for saying "oh yes", these symptoms occur with this disease
S12	Ich bin seit Jahren Ansprechpartnerin der Regionalgruppe ORT der SELBSTHILFEGRUPPE. Ich bilde mich also praktisch weiter durch Arztkongresse, die mir angeboten werden oder durch Veranstaltungen die ich jedes Jahr mit einem erfahrenen Arzt selber durchführe. Und dieses Wissen gebe ich weiter, im Moment finden auch viele Zoom-Veranstaltungen statt, durch Corona natürlich. Und vorher glaube ich kann können meine Mitglieder gut davon profitieren, oder die Leute, die mich	I have been the contact person for the regional group LOCAL of the PATIENT ORGANIZATION for years. Consequently, I keep myself informed through medical congresses that are offered to me or through events that I organize myself every year with an experienced doctor. And I pass on this knowledge, at the moment there are also many Zoom events, because of COVID of course. And before that, I think my members can benefit from it, or the people who call me not knowing where to go, which doctor knows what to do, and

	dann anrufen und nicht wissen wohin sie gehen können, welcher Arzt sich auskennt und und und. Das wollte ich jetzt bloß sagen.	so on. That's all I wanted to say.
S13	Ich hatte vorher vergessen zu sagen, dass auch eine Selbsthilfegruppe leite, die wir vor Jahren gegründet haben. Wir haben als Hilfestellung auf unserer Webseite eine Checkliste für den Arztbesuch erstellt, damit man die richtigen Fragen stellt, weil manche Fragen gar nicht gestellt werden, die sinnvollerweise beantwortet werden sollten. Und ich denke, das ist für den Ein- oder Anderen auch hilfreich, aber nur für ERKRANKUNGSGRUPPE.	I forgot to mention earlier that I also run a patient organization that we started years ago. We have created a checklist for the visit to the doctor on our website to help people ask the right questions, because some questions are not asked which should be answered. And I think this is also helpful for others, but only for the DISEASE GROUP.
S14	Bei mir ist es natürlich so, ich habe viel im Internet recherchiert und bin dann auf die Firma Novartis gekommen, von den Pharma Firmen. Und von denen musste ich sagen hab ich ganz viel Material an die Hand bekommen, über die Erkrankung und eben auch so eine Checkliste für den Arzt. Da sind schon die typischen Symptome für die Erkrankung vorgegeben und sogar immer noch von 1 bis 10, wie stark haben sie diese Symptome. Und das finde ich eigentlich sehr, sehr praktisch. Es ist wie es immer ist, man sitzt beim Arzt und es fällt einem nicht mehr ein.	In my case, of course, I did a lot of research on the internet and then came across the company Novartis, one of the pharmaceutical companies. And I have to say that I received a lot of material from them about the disease and also a checklist for the doctor. The typical symptoms of the disease are already given, and even from 1 to 10, how strong are these symptoms. And I actually consider that very, very practical. It is like it always is, you are sitting at the doctor's office and you just cannot think of anything else to say.
S15	Ja, gerade zu dem was PERSON gerade gesagt hat. Hilfreich wäre es zum Beispiel wenn man Flyer der	Yes, just to what PERSON has just said. It would be helpful, for example, if patient organization flyers could be displayed at

	Selbsthilfe bei den Ärzten auslegen dürfte und die Ärzte dafür auch etwas Interesse hätten. Zumindest sagen Ok sie dürfen die Flyer auslegen, auch wenn es selten ist. Es gibt vielleicht doch jemanden. Dies würde es auch schon einmal erleichtern und dann trotzdem auch diese Suche im Internet. Wo gibt es jemanden? Wo gibt es eine Selbsthilfegruppe? Wenn die Ärzte dafür offen wären, Hausärzte, die Neurologen in den Kliniken – da denke ich würde man auch die Leute erreichen.	the doctors' offices and if the doctors would be interested in them. At least say 'Ok you can distribute the flyers, even if it is rare'. There might be someone after all. Even then, it would facilitate the search on the internet. Where is there someone? Where to find a patient organization? If the doctors were open to it, family doctors, the neurologists in the clinics - I think you would also reach the people.
