# Supplementary material for: Identifying project topics and requirements in a citizen science project in rare diseases: a participative study
Source: Orphanet J Rare Dis. 2022 Sep 14;17:357. doi: 10.1186/s13023-022-02514-3 (PMC9476337; doi:10.1186/s13023-022-02514-3)
Supplement: Supplementary file 7 — Additional file 7: Topic prioritization. [file 13023_2022_2514_MOESM7_ESM.pdf]

Additional file 7: Topic prioritization

The following table shows the prioritization of the topics and the related derived (sub-) categories of both focus groups (1="most important", 2="very important" and 3="important") on average.

Topic	Category	Sub-Category	Voting Points average focus group 1	Voting Points average focus group 2
**Symptom tracking/Patient documentation support, symptom tracking**	A	A-I	3.0	2.0
**Overview of the previous (social) care**		A-I	1.0	-
**Self-managed patient file**		A-I	2.0	-
**Digitization of handwritten findings**		A-II	2.0	-
**Data collection for medical registries**		A-III	-	1.5
**Exchange of experience and networking**	B	B-I & B-II	2.3	1.25
**Checklists (for visits to the doctor)**		B-III	2.3	
**Information provision for medical professionals**		B-II		2.4
**Awareness-raising among service provider and visibility of RD**	C	C-I	2.0	2.3
**Acceleration of diagnosis**		C-II	1.8	-
**Improvement of guidelines**		C.III	3.0	-
